# Physiological fluid shear alters the virulence potential of invasive multidrug-resistant non-typhoidal *Salmonella* Typhimurium D23580

**DOI:** 10.1038/npjmgrav.2016.21

**Published:** 2016-06-09

**Authors:** Jiseon Yang, Jennifer Barrila, Kenneth L Roland, C Mark Ott, Cheryl A Nickerson

**Affiliations:** 1 Center for Infectious Diseases and Vaccinology, The Biodesign Institute, Arizona State University, Tempe, AZ, USA; 2 School of Life Sciences, Arizona State University, Tempe, AZ, USA; 3 Division of Biomedical Research and Environmental Sciences, NASA Johnson Space Center, Houston, TX, USA

## Abstract

*Salmonella enterica* serovar Typhimurium strains belonging to sequence type ST313 are a major cause of fatal bacteremia among HIV-infected adults and children in sub-Saharan Africa. Unlike “classical” non-typhoidal *Salmonella* (NTS), gastroenteritis is often absent during ST313 infections and isolates are most commonly recovered from blood, rather than from stool. This is consistent with observations in animals, in which ST313 strains displayed lower levels of intestinal colonization and higher recovery from deeper tissues relative to classic NTS isolates. A better understanding of the key environmental factors regulating these systemic infections is urgently needed. Our previous studies using dynamic Rotating Wall Vessel (RWV) bioreactor technology demonstrated that physiological levels of fluid shear regulate virulence, gene expression, and stress response profiles of classic *S*. Typhimurium. Here we provide the first demonstration that fluid shear alters the virulence potential and pathogenesis-related stress responses of ST313 strain D23580 in a manner that differs from classic NTS.

Recent epidemic outbreaks of multidrug-resistant invasive non-typhoidal *Salmonella* (NTS) in sub-Saharan Africa highlight the continual threat of emerging pathogens and reinforce the urgent need for new insight into the mechanisms by which these novel pathovars cause disease.^1^ Sequence analysis of D23580, a representative isolate belonging to the recently identified ST313 pathovar, revealed genome degradation resembling that of the human-restricted serovar Typhi.^1^ Combined with the highly invasive clinical presentation of bacteremia, these findings suggested that, although classified as NTS, D23580 might display a Typhi-like host tropism.^1^ Subsequent studies confirmed that D23580 still retains a broad host specificity that is characteristic of serovar Typhimurium, but also revealed the key pathogenesis characteristics that distinguish it from classic NTS.^2,3^

The pathogenicity of *Salmonella* can be altered in response to a variety of environmental conditions, including pH, temperature, oxygen, and nutrient availability.^4^ It has also become increasingly clear that physical/mechanical forces, including fluid shear, have an important role in regulating the virulence, gene expression, and/or pathogenesis-related stress responses of *Salmonella* and other bacteria.^5–8^ The NASA-engineered Rotating Wall Vessel (RWV) bioreactor is a suspension culture system that allows bacteria to grow under physiologically relevant low fluid shear (LFS) culture conditions (<0.01 dynes/cm^2^) when the reactor is oriented in the LFS orientation (Supplementary Figure 1). The LFS culture environment is disrupted when the bioreactor is adjusted to the higher fluid shear (HFS) orientation, as the sedimentation of cells, density gradients, and frictional and centrifugal forces increase the fluid shear as compared with LFS. Whereas the RWV was originally designed to simulate LFS conditions normally experienced by cells during culture in the quiescent environment of spaceflight, we have shown that these fluid shear levels are also relevant to those encountered by pathogens in the infected host, including the intestinal tract—the initial site of *Salmonella* infection.^5,9^ Moreover, *Salmonella* respond to these forces in novel ways that are directly relevant to the infectious disease process that cannot be observed using traditional shake and static flask cultures.^5^

It was previously demonstrated that LFS culture of *S.* Typhimurium strain χ3339 (an animal passaged-derivative of classic NTS strain SL1344) led to increased virulence, global changes in gene expression, and increased resistance to multiple pathogenesis-related stressors.^5,6^ It was subsequently shown that several other *Salmonella* serovars were able to sense and respond to alterations in fluid shear.^7^ A wide range of fluid shear levels are experienced by *Salmonella* in the environment and *in vivo*, with *in vivo* niches ranging from HFS in the bloodstream to LFS in between the brush border microvilli of epithelial cells.^10,11^ In addition, a correlation exists between LFS levels experienced by pathogens in the RWV and those naturally encountered in the infected host,^9^ including the intestinal tract. Accordingly, fluid shear is an important consideration when mimicking the biomechanical force microenvironment encountered by pathogens during infection, as conventional shake and static flasks often do not recapitulate these mechanical cues. As a facultative intracellular pathogen that incorporates both an intracellular and cell-free lifestyle, the spread of *Salmonella* throughout the gastrointestinal tract to the extraintestinal environment of the circulatory system exposes the pathogen to a broad range of fluid shear environments. Understanding how this important environmental signal can regulate the onset of disease and its progression is a critical consideration for the treatment and prevention of invasive salmonellosis by ST313 pathovars. Therefore, in this study we investigated the influence of physiological fluid shear on the virulence and several pathogenesis-related stress responses of the representative ST313 strain D23580.

D23580 was cultured to mid-to-late log phase in the RWV oriented in the LFS or HFS condition (Supplementary Figure 1) in Lennox Broth at 25 r.p.m. (rotations per minute) and 37 °C for 4 h. Growth curves were performed to ensure that cultures were profiled at identical phases of growth for all studies (Supplementary Figure 2). For virulence studies, 8-week-old female BALB/c mice were fasted for approximately 5 h and then perorally infected with increasing dosages of D23580 that were harvested immediately following RWV culture and prepared in buffered saline gelatin. Food and water were returned to mice 30 min after infection. Mice were monitored for 30 days. The 50% lethal dose (LD_50_) was calculated using the method of Reed and Muench^12^ using the results from three independent trials. The LD_50_ values obtained for the LFS and HFS groups were not significantly different, with 7.51×10^5^ and 6.53×10^5^ colony-forming units (CFUs), respectively. However, mice infected with D23580 cultured in the HFS condition exhibited more rapid disease progression, resulting in earlier time to death (Figure 1). This pattern was observed for dosages ranging from 10^4^ to 10^6^ CFU, with the group infected with 10^5^ CFU (approaching the LD_50_) displaying the biggest difference. Intriguingly, these results were the opposite of what was previously observed for classic NTS strain χ3339 using the same media, temperature, and similar growth phase, in which mice infected with χ3339 cultured in the LFS condition led to a more rapid time to death and decreased LD_50_.^5^

*Salmonella* encounters a number of stressors in the environment and during the course of infection, including acidic pH, oxidative, bile, and osmotic stresses. To evaluate the influence of fluid shear on the stress resistance of D23580, the strain was cultured in the RWV in the LFS and HFS orientations and subsequently subjected to acid (pH 3.5), bile salts (10%), oxidative (0.06% hydrogen peroxide), or osmotic (4 M NaCl) stress. Samples were plated on Lennox Broth agar at select time points to obtain CFU/ml, and the counts at each time point were normalized to the initial inoculum to obtain percent survival. Statistical significance between the LFS and HFS groups was determined using Student’s *t*-test (*α*=0.05). D23580 grown in the HFS condition exhibited increased resistance to killing by bile salts and H_2_O_2_-induced oxidative stress (Figures 2a and b) as compared with LFS cultures for the tested time points (*P*<0.05). These findings are in line with the decreased time to death in mice infected with the HFS condition. In addition, we also investigated the effect of fluid shear on the ability for D23580 to resist killing at pH 3.5 (Figure 2c) and 4 m NaCl (data not shown). However, no statistical differences were observed.

To our knowledge, this is the first report to demonstrate that physiological fluid shear regulates disease progression and pathogenesis-related stress responses for any strain belonging to the highly invasive ST313 pathovar. In response to culture under HFS conditions, D23580 exhibited a more rapid time to death in mice and displayed an increased resistance to bile salts and oxidative stress. These findings suggest that D23580 responds to fluid shear in a different manner than previously observed for classic NTS, and that HFS environments may enhance the resistance of this pathogen to environmental stress responses, which in turn may influence disease progression. Ongoing studies in our laboratory are focused on investigating the mechanisms underlying these differences. These results provide further evidence of the correlation between fluid shear and microbial mechanosensation, a rapidly emerging area of investigation that is unveiling novel insight into the role of mechanical forces in regulating microbial pathogenesis.

## Figures and Tables

**Figure 1:**
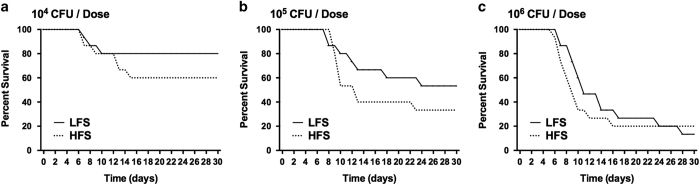
Survival of mice following peroral infection with D23580 grown in the Rotating Wall Vessel (RWV) under low fluid shear (LFS) or higher fluid shear (HFS). D23580 was cultured in the RWV under LFS or HFS to mid-to-late log phase in Lennox Broth (LB) media. Doses ranging from 10^2^ to 10^9^ colony-forming units (CFU) per mouse (five mice per dose) were administered to 8-week-old female BALB/c mice perorally. Mice were monitored three times a day for 30 days. An uninfected control group was included. (**a**–**c**) The time to death of mice infected with 10^4^, 10^5^, or 10^6^ CFU, respectively. Percent survival is defined as the percentage of mice surviving at the indicated number of days post infection. The median lethal dose (LD_50_) was determined by the method of Reed and Muench.^12^

**Figure 2:**
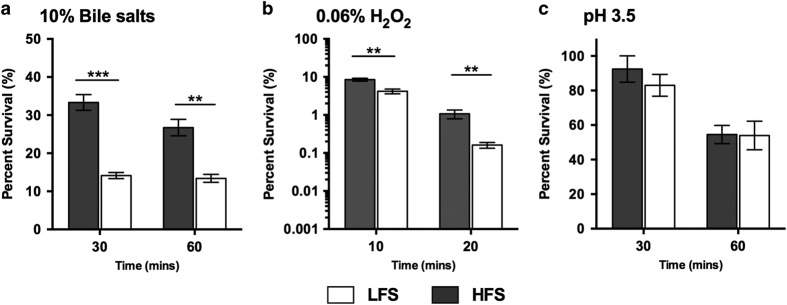
Resistance of Rotating Wall Vessel (RWV)-cultured D23580 to pathogenesis-related stresses. Bacterial cultures were grown in the RWV under low fluid shear (LFS; white bars) or higher fluid shear (HFS; gray bars), removed from reactors, and immediately subjected to the stress indicated. All tests were performed using a minimum of two independent biological replicates. (**a**) Bile stress was induced by the addition of a bile salt solution (Sigma-Aldrich, St. Louis, MO, B8756) to a final concentration of 10% in each culture. The results from a representative experiment are shown. (**b**) Oxidative stress was induced by adding hydrogen peroxide (H_2_O_2_) to a final concentration of 0.06% in the culture. Combined results from all trials are shown. (**c**) Acidic conditions were induced through the addition of a small pre-determined volume of citrate buffer (stock concentration of 1 m citrate, 0.513 m sodium phosphate dibasic heptahydrate) to lower the pH to 3.5. Combined results from all trials are shown. The pH was confirmed at the end of all experiments. For all stresses, samples were serially diluted in phosphate-buffered saline (PBS) and plated on Lennox Broth (LB) agar at time zero (T0, before the addition of stress) and at the indicated time points thereafter to determine the numbers of viable colony-forming units (CFU). Data were normalized at each time point to the number of initial bacteria subjected to the stress. The data are presented as the mean percent survival values with error bars indicating the Standard error of the mean. Statistical comparisons were made using Student’s *t*-test (***P<0.001; **P<0.01).
