# Supplementary figures and images for: Physiological fluid shear alters the virulence potential of invasive multidrug-resistant non-typhoidal Salmonella Typhimurium D23580
Source: NPJ Microgravity. 2016 Jun 9;2:16021–. doi: 10.1038/npjmgrav.2016.21 (PMC5515522; doi:10.1038/npjmgrav.2016.21)

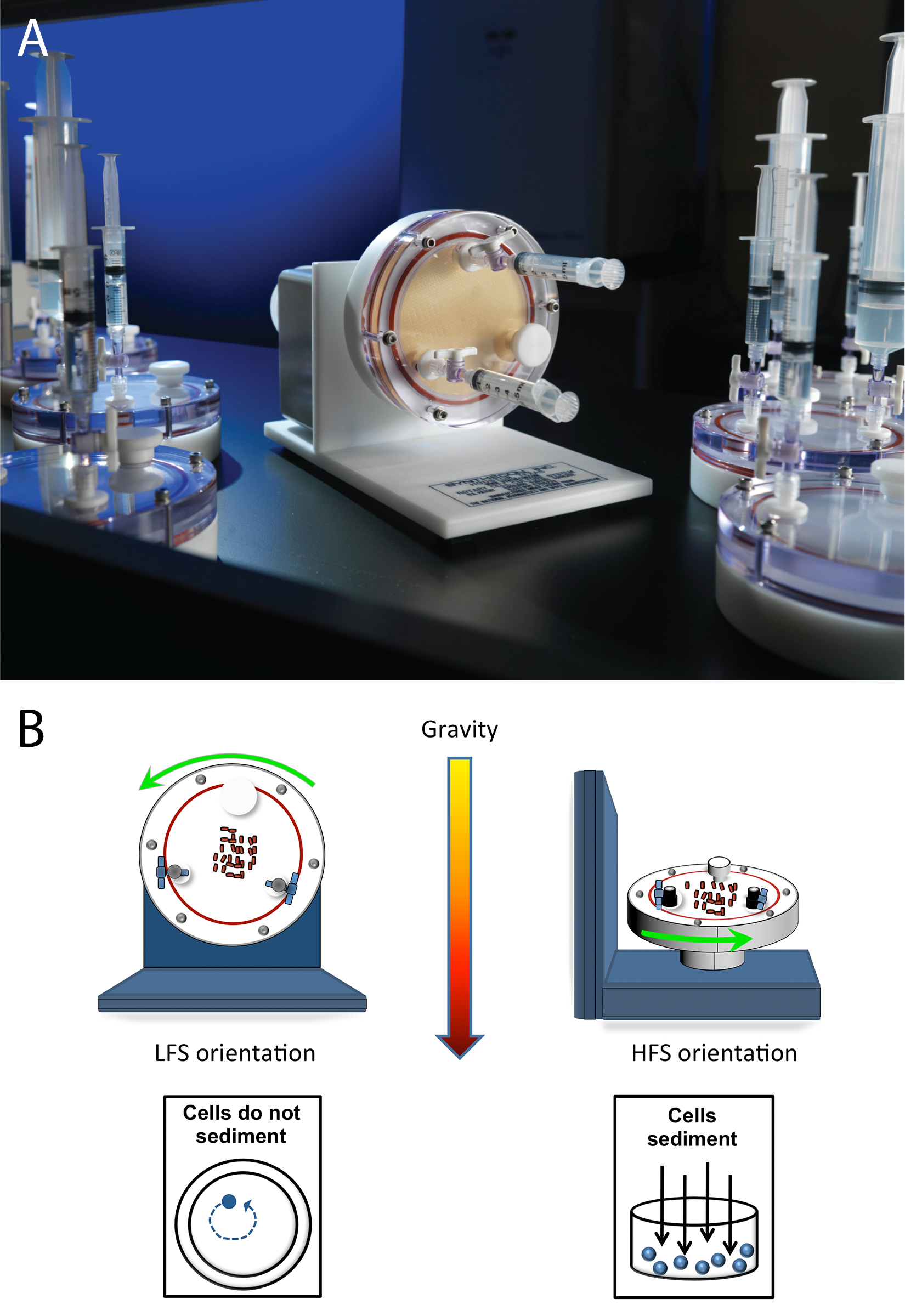

Supplement: Supplementary Figure 1 [file npjmgrav201621-s1.tiff]

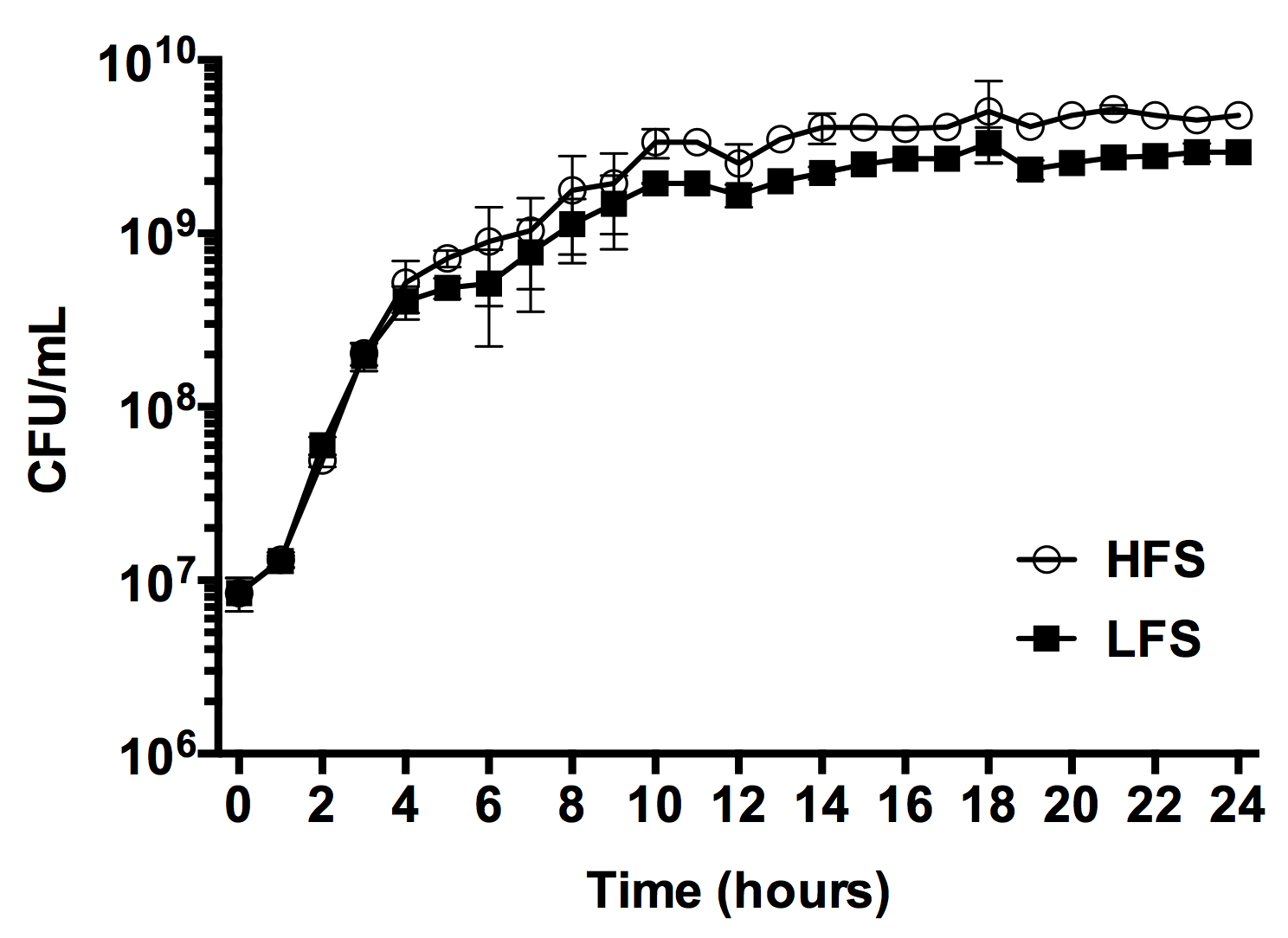

Supplement: Supplementary Figure 2 [file npjmgrav201621-s2.tiff]
